# Pre-mRNA splicing modulates post-harvest deterioration of cassava storage root

**DOI:** 10.1093/plphys/kiae294

**Published:** 2024-05-22

**Authors:** Yee-Shan Ku

**Affiliations:** Assistant Features Editor, Plant Physiology, American Society of Plant Biologists; School of Life Sciences and Centre for Soybean Research of the State Key Laboratory of Agrobiotechnology, The Chinese University of Hong Kong, Hong Kong SAR, China

With a storage root rich in carbohydrates, casava is a major food crop in many tropical and subtropical regions; in addition to providing human food, the root also serves as the raw material of biopolymers and cosmetic products (Howeler et al. 2013; Li et al. 2017). However, during harvesting, the root is inevitably damaged. The wounding triggers an oxidative burst and reactive oxygen species (ROS) accumulation, which leads to deterioration of the root (Zainuddin et al. 2018). Such postharvest physiological deterioration (PPD) limits the consumption of cassava. Understanding the PPD mechanism will help develop strategies to increase the shelf life of cassava storage root after harvesting.

In this issue of *Plant Physiology*, Gu et al. identified genes that change expression in cassava storage root after harvesting (Gu et al. 2024). Interestingly, a portion of the differentially expressed genes also exhibited differential alternative splicing. The alternatively spliced genes include those encoding serine/arginine-rich (SR) proteins, which are RNA splicing regulators. SR proteins have conserved RNA-binding domains and have been shown to be involved in the assembly of spliceosome complex. The alternative splicing of SR protein pre-mRNAs results in proteins with different isoforms, which have diverse functions in the alternative splicing of other transcripts (Reddy and Shad Ali 2011). Thus, these results hint at the possible role of SR protein–mediated alternative splicing in regulating PPD.

Because ROS accumulation is closely related to PPD (Wahengbam et al. 2023), the authors tested the splicing patterns of ROS scavenging-related genes during PPD (Gu et al. 2024). Specifically, they looked at candidate genes, including *MeGPX4*, *MeGST18*, and *MeGSH2*, which encode glutathione peroxidase, glutathione transferase, and glutathione synthetase, respectively. Expression analyses revealed that these genes are alternatively spliced after harvesting. These results hinted at the regulation of PPD through modulating ROS accumulation. Further data mining revealed that the genes exhibiting both differential expression and differential alternative splicing were enriched in biological processes, including RNA splicing regulation, spliceosome complex assembly, and ABA stimulus. Specifically, ABA biosynthesis-related genes such as *MeABA1* and *MeABA2* were alternatively spliced with reduced expressions during PPD. The authors then showed that ABA treatment decreased ROS accumulation in the cassava storage root and alleviated PPD (Gu et al. 2024). Using cassava varieties with different PPD rates, the level of intron-retained *MeABA1* transcript was positively correlated to the PPD rate. As splicing factors, SR proteins possibly regulate the splicing of *MeABA1*.

Previously, the authors characterized the SR protein family in cassava (Gu et al. 2020). In this new work, they tested whether these proteins could bind to the pre-mRNA of *MeABA1* using yeast 3-hybrid and RNA immunoprecipitation assays. They further characterized 1 of these SR protein putative splicing factors, MeSCL33 (SC35-like splicing factor 33). When *MeSCL33* was overexpressed, the intron-retained transcript level of *MeABA1* was decreased, and the fully spliced transcript level of *MeABA1* was increased, demonstrating a role for MeSCL33 in the splicing of MeABA1. Moreover, in the *MeSCL33* overexpressors, ABA levels were increased, ROS accumulation decreased, and the rate of postharvest physiological decay was decreased.

**Figure 1.**
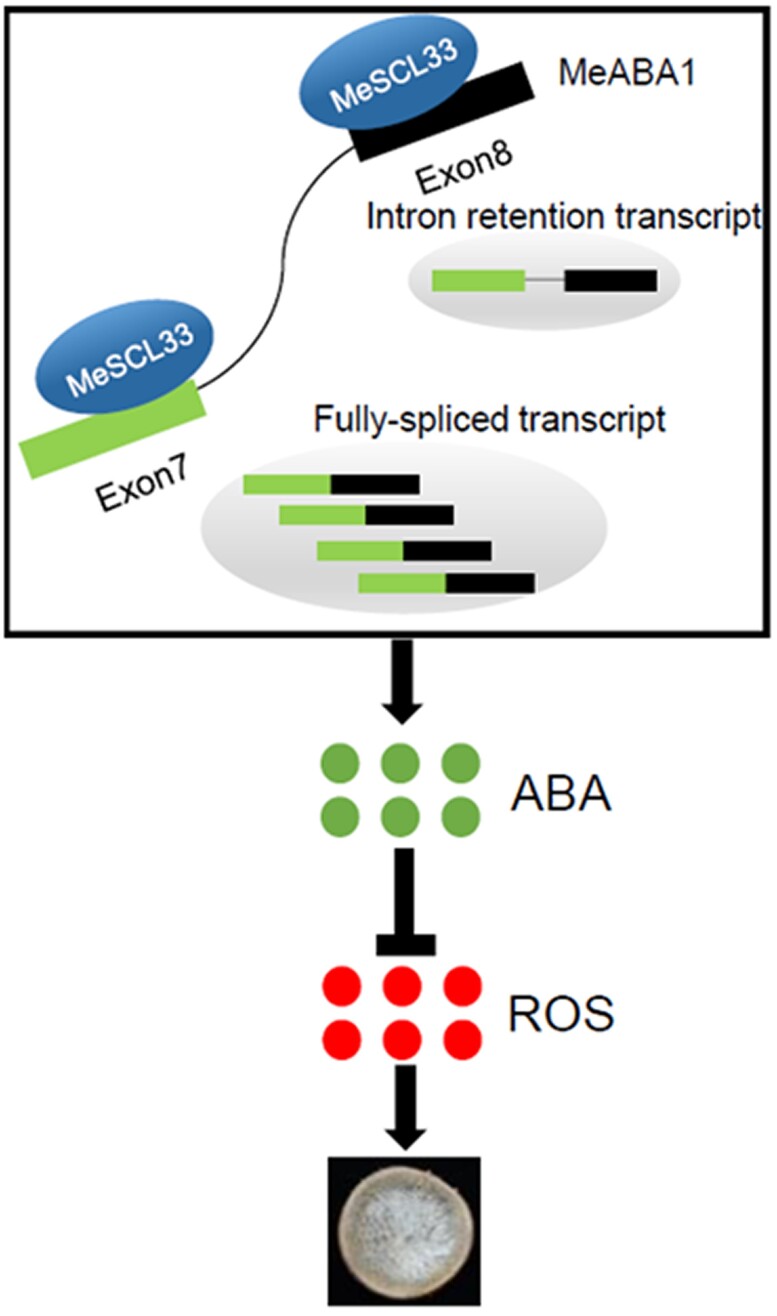
During PPD of cassava storage root, MeSCL3 binds to the pre-mRNA of *MeABA1* and promotes its proper splicing. The fully spliced *MeABA1* transcripts result in enhanced biosynthesis of ABA, which reduces ROS accumulation and alleviates PPD of cassava root. The figure is adopted from Gu et al. (2024).

In summary, the harvesting of cassava storage root creates wounds and results in ROS accumulation, which promotes PPD. However, the ROS accumulation and the enhanced deterioration can be alleviated by ABA. This study uncovers the role of ABA in regulating cassava storage root PPD. In addition, the authors report the role of the splicing factor MeSCL33 in promoting the level of fully spliced *MeABA1* transcript, which leads to elevated ABA levels, decreased ROS accumulation, and alleviated PPD. The proposed mechanism of MeSCL3-mediated alleviation of PPD in cassava storage root is summarized in Fig. 1 (Gu et al. 2024).
